# Laser Desorption/Ionization Mass Spectrometry as a Potential Tool for Evaluation of Hydroxylation Degree of Various Types of Titanium Dioxide Materials

**DOI:** 10.3390/ma14226848

**Published:** 2021-11-13

**Authors:** Małgorzata Kasperkowiak, Monika Kurowska, Maciej Zalas, Rafał Frański

**Affiliations:** 1 Center for Advanced Technology, Adam Mickiewicz University, Uniwersytetu Poznańskiego 10, 61-614 Poznań, Poland; malgorzata.kasperkowiak@amu.edu.pl; 2 Faculty of Chemistry, Adam Mickiewicz University, Uniwersytetu Poznańskiego 8, 61-614 Poznań, Poland; monkur4@st.amu.edu.pl (M.K.); maciej.zalas@amu.edu.pl (M.Z.)

**Keywords:** titanium dioxide, laser desorption/ionization, mass spectrometry, surface analysis, hydroxyl group

## Abstract

For many applications, TiO_2_ must have a unique surface structure responsible for its desirable physicochemical properties. Therefore the fast and easy methods of TiO_2_ surface characterization are of great interest. Heated TiO_2_ samples and dye-modified TiO_2_ samples were analyzed by laser desorption/ionization mass spectrometry. In the negative ion mode, two types of ions were detected, namely (TiO_2_)_n_^−^ and (TiO_2_)_n_OH^−^. It has been established that the samples can be differentiated based on the relative ion abundances, especially with respect to the free hydroxyl group population. It indicates that laser desorption ionization mass spectrometry has the potential for the investigation of the surface properties of various TiO_2_ materials.

## 1. Introduction

TiO_2_ is one of the world’s most common material, widely used in many fields of science and branches of industry as well as in everyday life. For example, it has been applied as a pigment (e.g., in toothpaste [1]), as semiconductors (e.g., in solar cells [2]), as catalysts (e.g., in biodiesel production [3]), as gas sensors (e.g., as alcohol vapor sensor [4]), etc.

For many of its applications, TiO_2_ must have a unique surface structure that provides desirable physicochemical properties of TiO_2_, necessary for its effective use. One of the most important features of the TiO_2_ surface is the number and distribution of the hydroxyl groups present on it [5,6,7,8,9]. For example, it has been demonstrated and discussed in detail, that the surface hydroxylation degree is of crucial importance for selective adsorption of Cr(VI) [10], photocatalytic oxidation/degradation of methyl ethyl ketone [11], phenolphthalein [12], methyl orange, rhodamine B, *p*-chlorophenol [13], methylene blue [14], adsorption of CO_2_, SO_2_, NO_2_ [15]. The most common method for the study of surface hydroxylation degree, or more precisely the tool which enables comparison of the TiO_2_ hydroxylation degrees, is the Fourier-transform infrared spectroscopy (FTIR) [8,9,10,11,12,13,14,15], which sometimes is supported by X-ray photoelectron spectroscopy (XPS), temperature-programmed desorption or surface acid–base ion-exchange reactions method [7,10,13].

A specific TiO_2_ application is in the mass spectrometry, namely as a substrate (solid matrix) in the surface-assisted laser desorption/ionization mass spectrometry [16,17,18,19]. There are also a number of papers reporting TiO_2_ surface modification or generation of interesting gas-phase titanium-oxide clusters by subjectingTiO_2_ surface to laser beam action [20,21,22,23,24]. The goal of this work is to check if laser desorption/ionization mass spectrometry (LDI-MS) may be used for TiO_2_ surface characterization, namely to evaluate the TiO_2_ hydroxylation degree. For this purpose, the samples of TiO_2_ prepared at different temperatures and dye-modified TiO_2_ samples have been analyzed by LDI-MS.

## 2. Materials and Methods

Samples of heated TiO_2_ were prepared by solvothermal hydrolysis of titanium tetraisopropoxide (Aldrich, Poznań, Poland) and heating (calcination) of the obtained material at different temperatures (100, 200, 300, 400, 500 and 600 °C) according to the procedure described elsewhere [25]. The obtained samples were characterized by FT-IR spectra recorded on an IFS-66/s spectrometer (Bruker, Billerica, MA, USA) using KBr powder as a diluent.

**Scheme 1:**
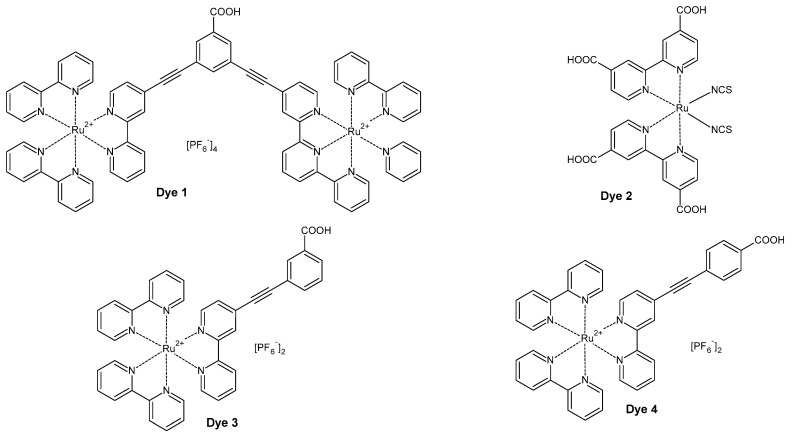
Structures of dyes used for modification of P25 TiO_2_.

The dye-modified TiO_2_ samples were prepared from commercially available P25 TiO_2_ Aeroxide (Evonik, Essen, Germany) by using four different dyes (Scheme 1) according to the procedure described elsewhere [26,27,28].

The LDI TOF MS (laser desorption/ionization time of flight mass spectrometry, was used to generate the gas-phase clusters. Portions of 1 µL of TiO_2_ suspension in methanol, were spotted onto the target (MTP 384 ground steel, Bruker Daltonics, Bremen, Germany). During MS experiments, an Ultraflex TOF/TOF spectrometer (Bruker Daltonics, Bremen, Germany) was operated in reflection mode (both positive and negative) in the range of *m*/*z* 50–1500. For *m*/*z* > 1000, the clusters were characterized by low intensity. This spectrometer is equipped with a SmartBeam II laser (λ = 355 nm). Metastable fragmentation of selected ions was induced by laser without further use of collision gas. The LIFT technology was employed [29]. The software flexControl v.3.4 was used for data acquisition and collection, whereas flexAnalysis v.3.4 was used for data manipulation, evaluation, and processing.

## 3. Results and Discussion

**Figure 1:**
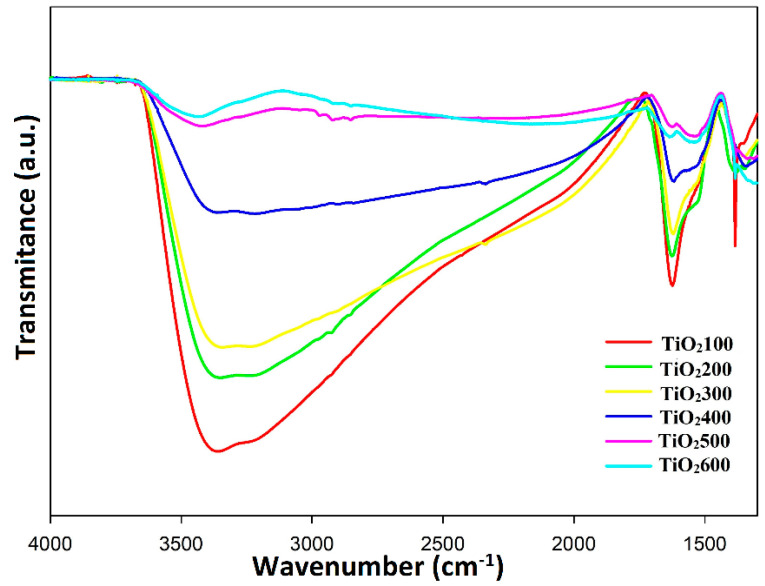
FTIR spectra of TiO_2_ samples.

Figure 1 shows the FTIR spectra of heated TiO_2_ samples. The intensities of the absorption bands at about 3400 and 1630 cm^−1^ indicate that the number of hydroxyl groups decreases in the order TiO_2_100 > TiO_2_200 > TiO_2_300 >> TiO_2_400 >> TiO_2_500 ≅ TiO_2_600.

The heating of TiO_2_ affects not only the hydroxylation degree, but also a number of other surface parameters, e.g., surface area, pore-volume, surface morphology, oxygen and titanium vacancies, surface roughness, surface micro-topography, etc. [30,31,32,33,34]. These parameters have a minor influence on the FTIR spectra, but may significantly affect the LDI mass spectra (which is easy to justify). Furthermore, the ions observed under LDI conditions are not only a result of the laser desorption/ionization process but also of the processes which occur in the laser plume containing energetic ions, neutrals, and electrons (in this hot cloud of particles, a number of gas-phase reactions can occur). Therefore, the key question is if moderate differences in the surface chemical composition, e.g., the hydroxylation degree, are reflected on the LDI mass spectra.

**Figure 2:**
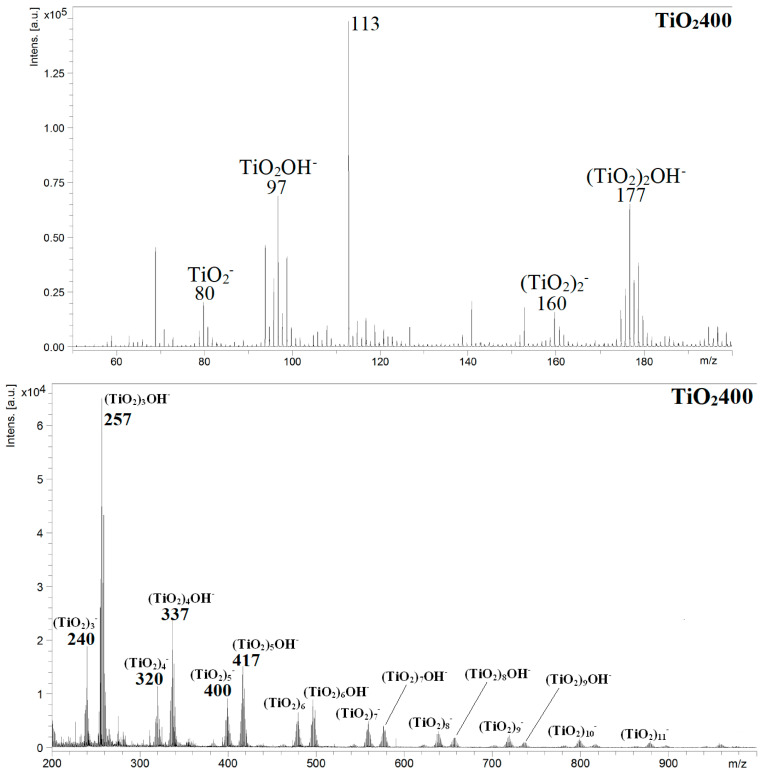
LDI mass spectra of TiO_2_400 obtained in the negative ion mode, in the *m*/*z* range 50–200 and 200–1000.

Figure 2 shows the LDI mass spectrum of TiO_2_400, obtained in the negative ion mode, in the *m*/*z* range 50–200 and 200–1000 (for clarity), as a representative example. The other LDI mass spectra are shown in the Supplementary Material (Figures S1 and S2). Thanks to the characteristic isotope signals (e.g., Figure S3), the titanium-containing ions can be easy identified and the lack of the characteristic isotope signals of titanium indicates that we deal with background/contaminant ions (e.g., that at *m*/*z* 113 most probably corresponds to [(HCOO)_2_Na]^−^, since formic acid is a common contamination of methanol, other contaminant ions may be PO_3_^−^ at *m*/*z* 79, CH_3_SO_3_^−^ at *m*/*z* 95, H_2_PO_4_^−^ and HSO_4_^−^, both at *m*/*z* 97, [35]). Two types of titanium-containing ions were identified, namely (TiO_2_)_n_^−^ and (TiO_2_)_n_OH^−^ (the formers are open-shell ions). It is clearly seen that with increasing n, we deal with a decrease in the (TiO_2_)_n_OH^−^/(TiO_2_)_n_^−^ ratio. It may be justified by the relative electron affinities of (TiO_2_)_n_OH and (TiO_2_)_n_ [5]. The key question is if the observed relative abundances of the titanium-containing ions depend on the surface properties of TiO_2_ samples subjected to the LDI process. The presence of OH-containing ions suggests that it may be possible to correlate the ion abundances with the hydroxylation degree of the TiO_2_ surface.

**Figure 3:**
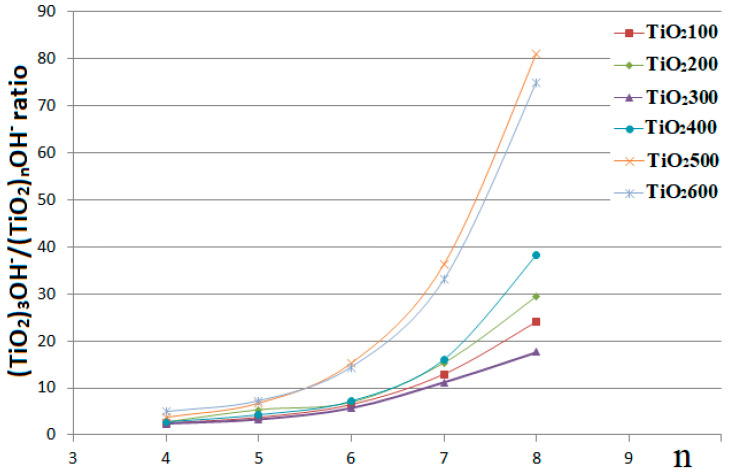
The breakdown plots of the (TiO_2_)_3_OH^−^/(TiO_2_)_n_OH^−^ ratio against the n number obtained for heated TiO_2_ samples..

The heating of the TiO_2_ results in an increase of particle size [25]; thus, it is expected that for TiO_2_ samples heated at higher temperatures, the ion abundances at a higher *m*/*z* range will be higher. However, the opposite situation was observed. It is visible that for TiO_2_ samples heated at higher temperatures, the ion abundances at a higher *m*/*z* range are lower (Figure S2). Thus, it is plausible that this phenomenon may depend on the surface hydroxylation degree. Figure 3 shows the breakdown plots of the (TiO_2_)_3_OH^−^/(TiO_2_)_n_OH^−^ ratio against the n number ((TiO_2_)_3_OH^−^ is an abundant ion at *m*/*z* 257, (the ions TiO_2_OH^−^ and (TiO_2_)_2_OH^−^ have similar abundances as (TiO_2_)_3_OH^−^). Except for the TiO_2_300 plot, the obtained plots, shown in Figure 3, reflect very well the hydroxylation degree, analogically as the FTIR spectrum shown in Figure 1.

It is worth adding that sometimes the desired specific properties of TiO_2_ can be obtained by its calcination at a given temperature. For example, the highest photocatalytic activity of TiO_2_ calcinated at 300 °C was attributed to the fact that at 300 °C the best trade-off between a few surface properties (including surface hydroxyl groups population) was reached [11]. In our case (Figure 3), it is clear that the surface properties of TiO_2_300 are responsible for the specific plot which deviates from the others.

**Figure 4:**
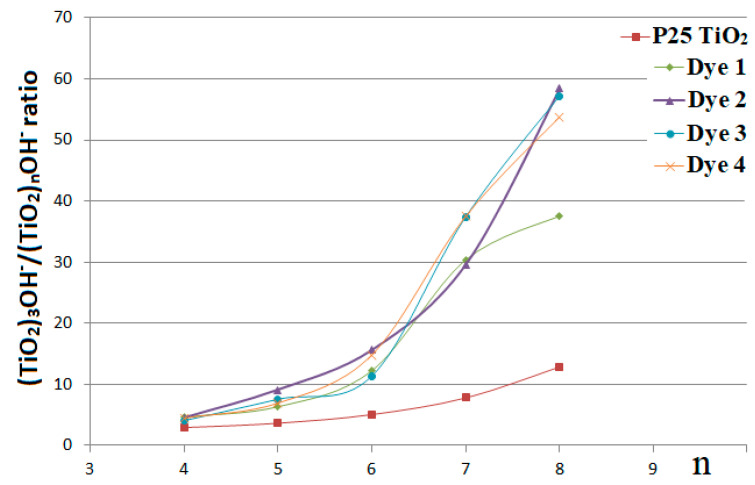
The breakdown plots of the (TiO_2_)_3_OH^−^/(TiO_2_)_n_OH^−^ ratio against the n number obtained for the unmodified P25 TiO_2_ and dye-modified samples.

It can be taken for granted that the dye-modified TiO_2_ samples have similar free surface hydroxyl group populations and they differ only in the dye structures. Figure S4 (Supplementary Material) shows the LDI mass spectra of the unmodified P25 TiO_2_ and dye-modified samples and Figure 4 shows the breakdown plots of (TiO_2_)_3_OH^−^/(TiO_2_)_n_OH^−^ ratio against the n number obtained for the unmodified P25 TiO_2_ and dye-modified samples.

It is clear that dye-modification yielded similar changes in the course of the plots as heating (Figure 3 and Figure 4). Namely, a substantial increase in the (TiO_2_)_3_OH^−^/(TiO_2_)_n_OH^−^ ratio was observed for n ≥ 6. Furthermore, the dye structures have minor (or moderate at most) influences on the character of the plots.

We also checked if the (TiO_2_)_n_OH^−^/(TiO_2_)_n_^−^ ratio depends on the hydroxylation degree. The obtained plots, shown in the Supplementary Material (Figures S5 and S6), can be used for the comparison/differentiation of the TiO_2_ samples; however, the comparison of the hydroxylation degrees has to be performed with caution as briefly discussed in the Supplementary Material.

It was also found that the TiO_2_^−^/(TiO_2_)_3_OH^−^ ratio also may depend on the hydroxylation degree, as shown in the Supplementary Material, Figure S7. It may be argued that the abundances of ions at the low *m*/*z* range (TiO_2_^−^ at *m*/*z* 80) may be affected by background/contaminant ions. However, it is plausible that the obtained TiO_2_^−^/(TiO_2_)_3_OH^−^ ratios reflect well the hydroxylation degree, especially for heated TiO_2_ samples (Figure S7). Furthermore, in contrast to the (TiO_2_)_3_OH^−^/(TiO_2_)_n_OH^−^ ratio, the ratio TiO_2_^−^/(TiO_2_)_3_OH^−^ does not show any exceptional behavior of TiO_2_300. Therefore, Figure S7 can be a good supplement to the plots shown in Figure 3 and Figure 4.

We also obtained the LDI mass spectra in the positive ion mode (Supplementary Material, Figure S8), but they were found to be useless for the purpose of this work. The ions (TiO_2_)_n_^+^ and TiO(TiO_2_)_n_^+^ were detected but in low abundance. Additionally, a number of abundant non-Ti-containing ions was detected as well (background/contamination).

There is a number of papers devoted to the gas-phase studies of neutral and charged titanium oxide clusters [23,24,36,37,38,39,40,41,42], since they provide some insight into the properties of bulk titanium oxide at the molecular level. Therefore, the (TiO_2_)_n_^−^ and (TiO_2_)_n_OH^−^ ions were subjected to further analysis, to get the spectra of metastable ions (LIFT mass spectra). The gas-phase metastable decompositions of (TiO_2_)_n_^−^ ions were found to be trivial, namely, the loss of TiO_2_ molecule occurred (Supplementary Material, Figure S9). The gas-phase metastable decompositions of (TiO_2_)_n_^−^ ions were trivial; namely, the loss of the TiO_2_ molecule occurred (Supplementary Material, Figure S9). It has been reported that (TiO_2_)_n_^−^ ions are very reactive towards trace of gases inside the instrument and are not prone to lose TiO_2_ molecule [23,24]. However, in tandem mass spectrometry, the gas-phase cluster ions may have different structures and dissociation behaviors [23]. The results obtained for (TiO_2_)_n_OH^−^ ions were quite surprising. In the obtained LIFT mass spectra, we detected the (TiO_2_)_n_H_2_O^−^ ions (Supplementary Material, Figure S10). Just to note, there is no doubt that in the full scan mass spectra, we deal with (TiO_2_)_n_OH^−^ ions, not with (TiO_2_)_n_H_2_O^−^ ions (Supplementary Material, Figure S3).

It is difficult rationalize how the (TiO_2_)_n_H_2_O^−^ ions can be formed from (TiO_2_)_n_OH^−^ ions. Most probably, at first (TiO_2_)_n_OH^−^ ions lose the OH^●^ radical producing (TiO_2_)_n_^−^ ions. Such a process has already been observed for (TiO_2_)_3_OH^−^ ion [24]. We are aware that the occurrence of this process may be disputable since it is the formation of two odd-electron species from the even-electron ion. Furthermore, in mass spectrometry, OH^●^ radical loss is not a favored process, even in electron ionization conditions. The produced (TiO_2_)_n_^−^ ions undergo two processes: losses of TiO_2_ molecules and reactions with traces of water inside the mass spectrometer, and the processes may occur in any order. In other words, in our experiments, the (TiO_2_)_n_^−^ ions formed under LIFT conditions are very reactive towards gas-phase water impurities, in contrast to the (TiO_2_)_n_^−^ ions produced in the LDI source. It must be added that our results are not contrary to those reported in [23,34], which have been obtained in different conditions (e.g., with respect to the reaction time). On the other hand, it indicates that the behavior of the gas-phase titanium oxide cluster is very sensitive to the conditions used.

## Data Availability

Data are available on request at corresponding authors.

## Supplementary Materials

The following are available online at https://www.mdpi.com/article/10.3390/ma14226848/s1, Figure S1: LDI mass spectra of TiO_2_ samples, obtained in the negative ion mode, in the *m*/*z* range 50–200. Figure S2: LDI mass spectra of TiO_2_ samples, obtained in the negative ion mode, in the *m*/*z* range 200–1000. Figure S3: The obtained isotope pattern of (TiO_2_)_7_OH^−^ ion, shown as a representative example. Figure S4: LDI mass spectra of the unmodified P25 TiO_2_ and dye-modified samples. Figure S5: The breakdown plots of the (TiO_2_)_n_OH^−^/(TiO_2_)_n_^−^ ratio against the n number obtained for heated TiO_2_ samples. Figure S6: The breakdown plots of the (TiO_2_)_n_OH^−^/(TiO_2_)_n_^−^ ratio against the n number obtained for unmodified P25 TiO_2_ and dye-modified samples. Figure S7: The TiO_2_^−^/(TiO_2_)_3_OH^−^ ratios obtained for heated TiO_2_ samples, unmodified (P25 TiO_2_) and dye-modified samples. Figure S8: LDI mass spectra of TiO_2_ samples obtained in the positive ion mode. Figure S9: Exemplary LIFT mass spectra of (TiO_2_)_n_^−^ ions. Figure S10: Exemplary LIFT mass spectra of (TiO_2_)_n_OH^−^ ions.
