# Supplementary material for: Laser Desorption/Ionization Mass Spectrometry as a Potential Tool for Evaluation of Hydroxylation Degree of Various Types of Titanium Dioxide Materials
Source: Materials (Basel). 2021 Nov 13;14(22):6848. doi: 10.3390/ma14226848 (PMC8619251; doi:10.3390/ma14226848)
Supplement: Supplementary file 1 [file materials-14-06848-s001.zip › materials-1408989-supplementary.pdf]

materials

MDPI

Supplementary Material

Laser Desorption/Ionization Mass Spectrometry as a Potential Tool for Evaluation of Hydroxylation Degree of Various Types of Titanium Dioxide Materials

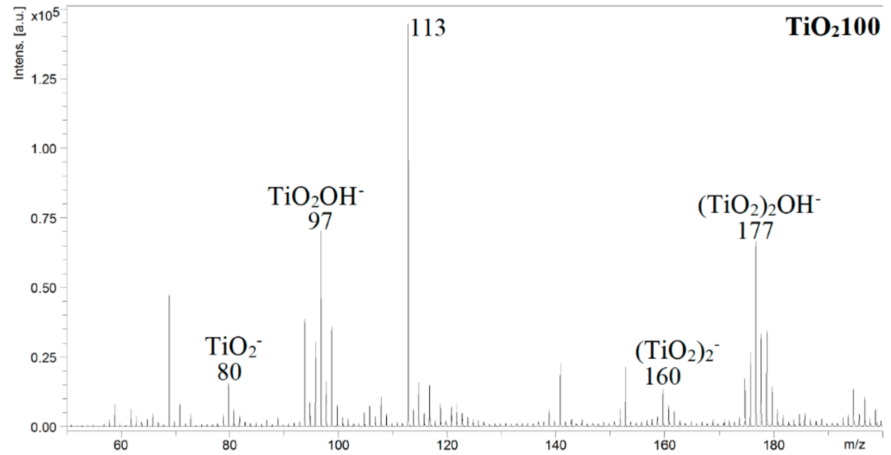

(a)

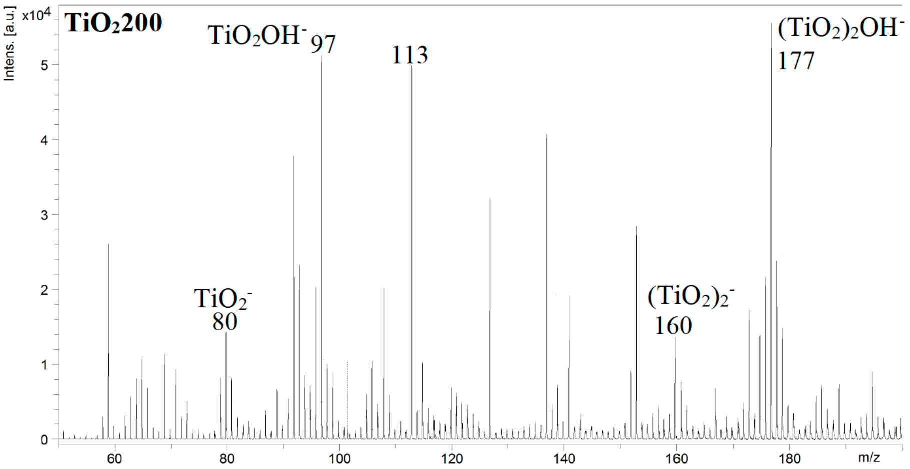

(b)

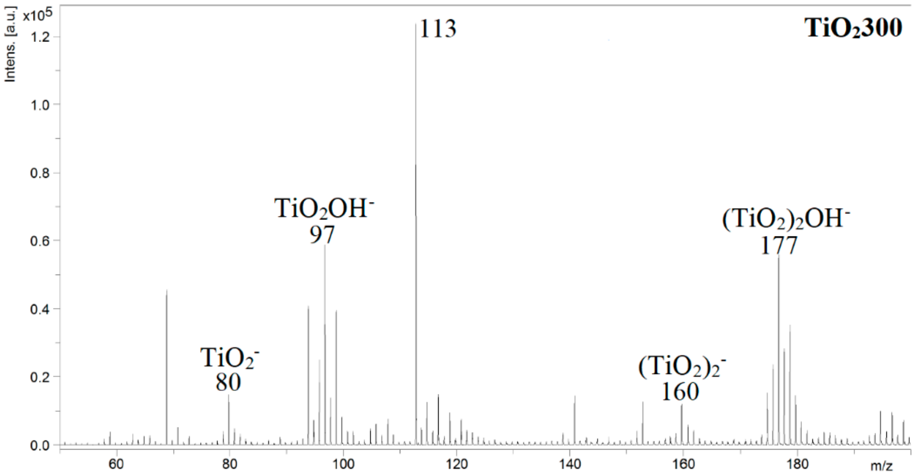

(c)

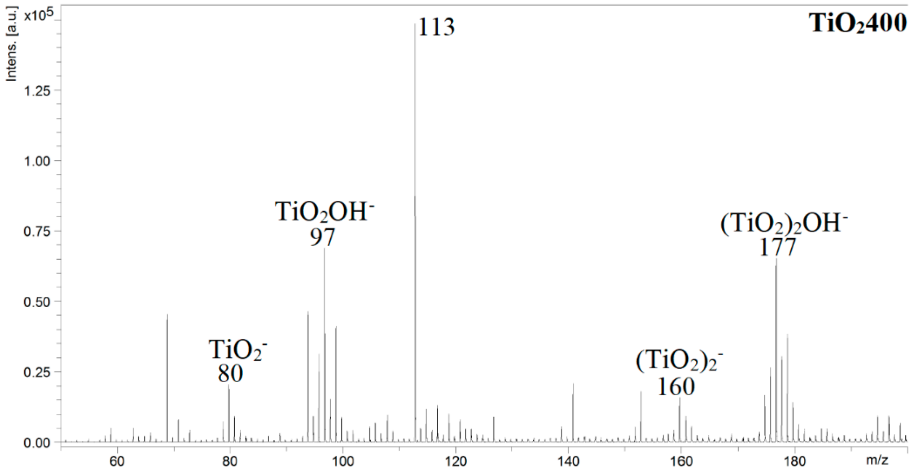

(d)

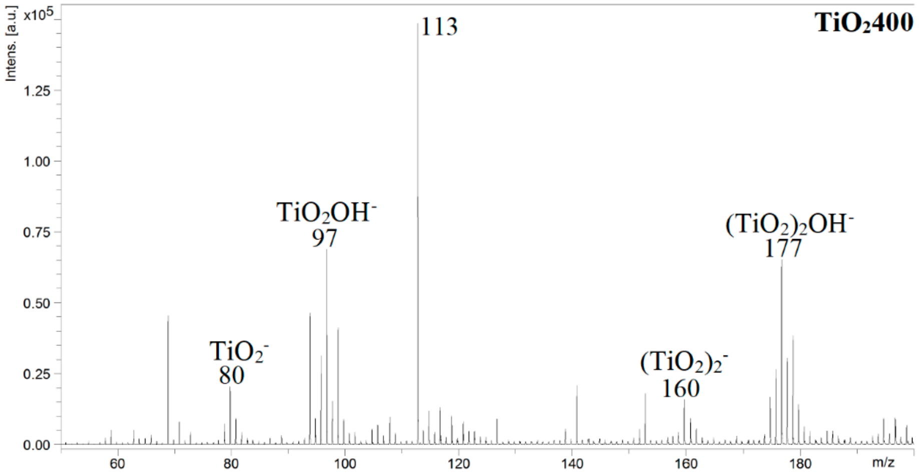

(e)

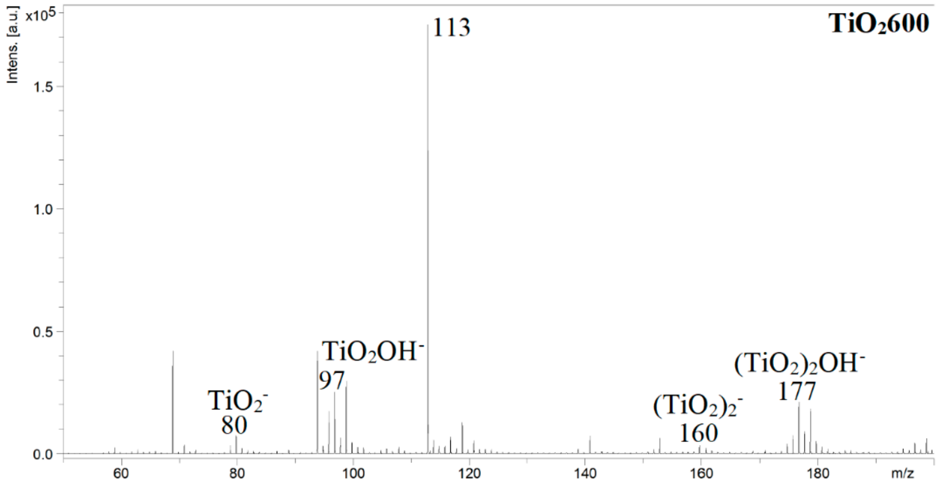

(f)

Figure S1. LDI mass spectra of TiO_2 samples, obtained in the negative ion mode, in the *m*/*z* range 50-200, (a) $TiO_2$100, (b) $TiO_2$200, (c) $TiO_2$300, (d) $TiO_2$400, (e) $TiO_2$500, (f) $TiO_2$600.

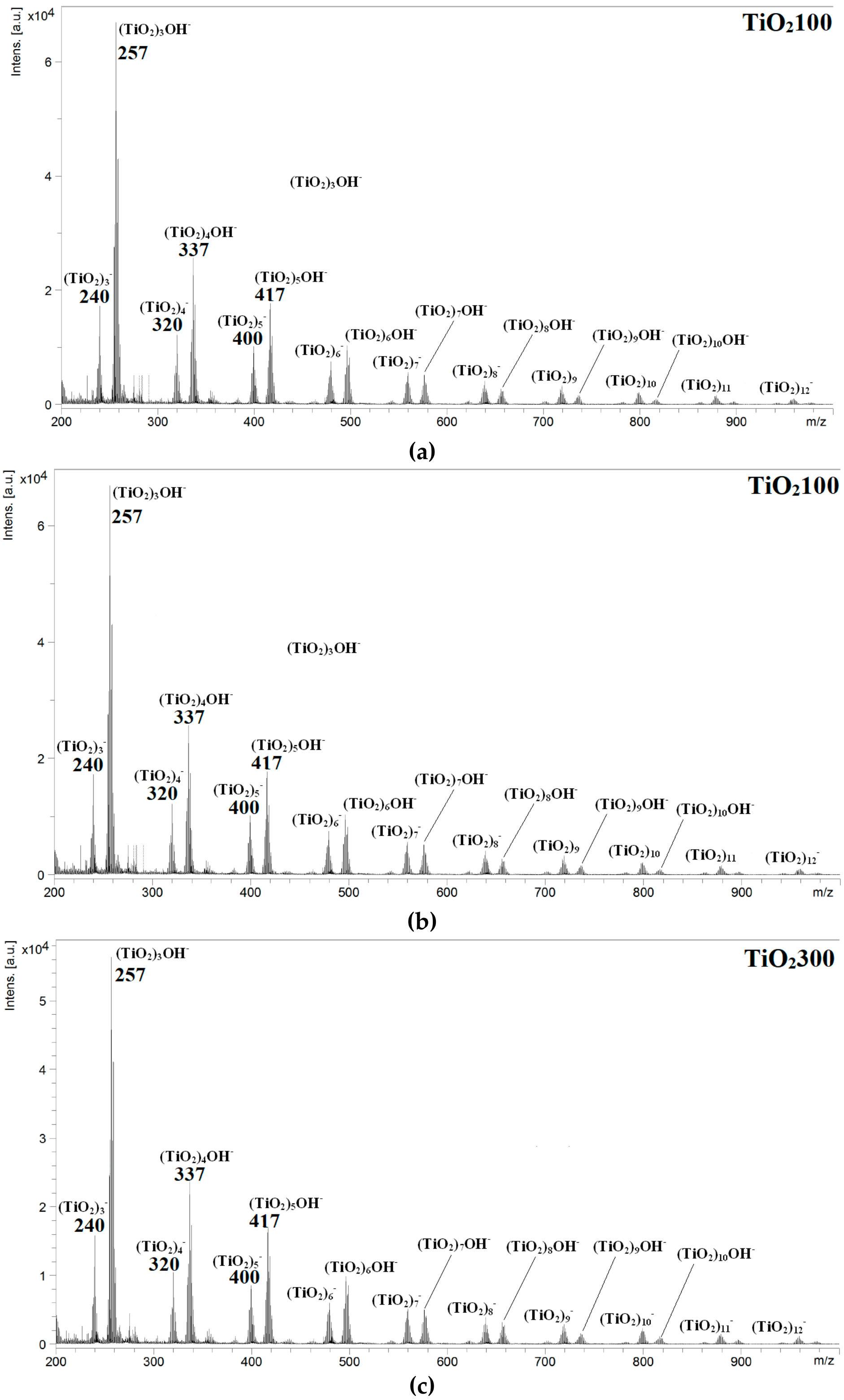
TiO2100
Intens. [a.u.]
x10^4
m/z
$(TiO_2)_3OH^-$ 257
$(TiO_2)_4OH^-$ 337
$(TiO_2)_5OH^-$ 417
$(TiO_2)_3^-$ 240
$(TiO_2)_4^-$ 320
$(TiO_2)_5^-$ 400
$(TiO_2)_6^-$
$(TiO_2)_6OH^-$
$(TiO_2)_7^-$
$(TiO_2)_7OH^-$
$(TiO_2)_8^-$
$(TiO_2)_8OH^-$
$(TiO_2)_9$
$(TiO_2)_9OH^-$
$(TiO_2)_{10}$
$(TiO_2)_{10}OH^-$
$(TiO_2)_{11}$
$(TiO_2)_{12}^-$
(a)
TiO2100
(b)
TiO2300
(c)

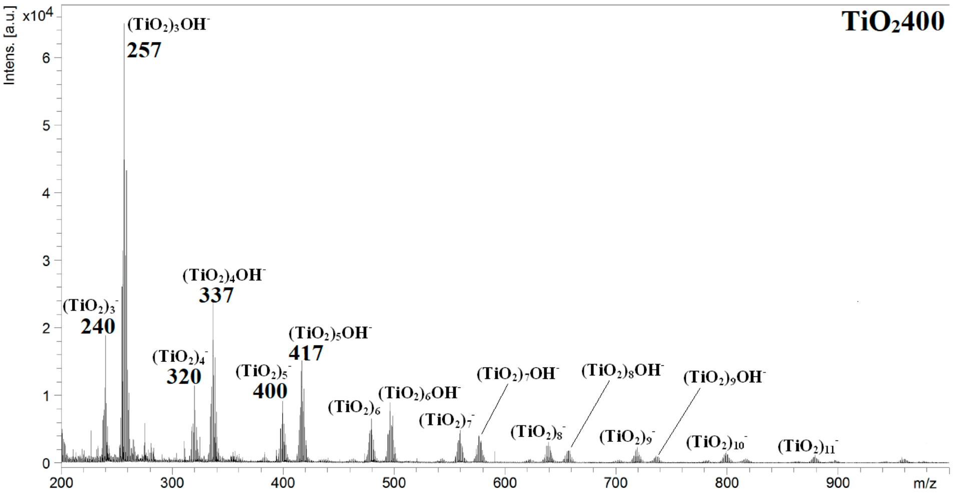

(d)

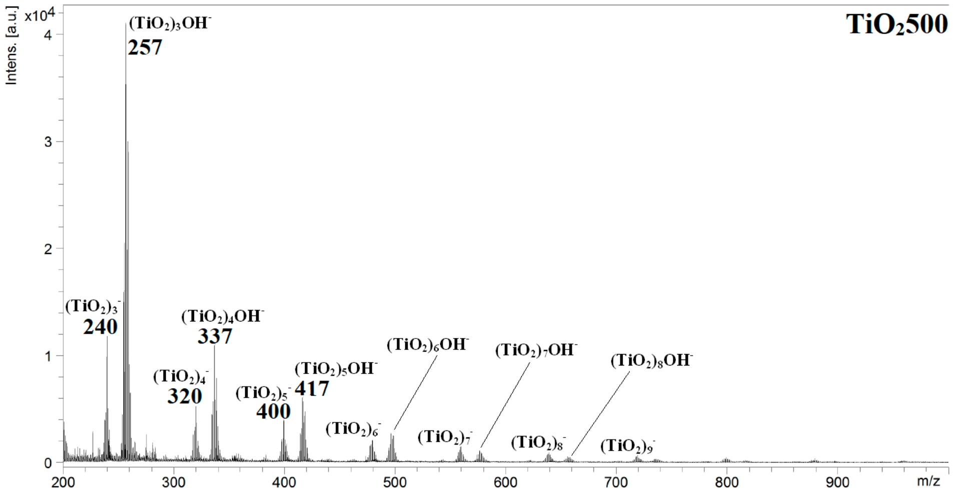

(e)

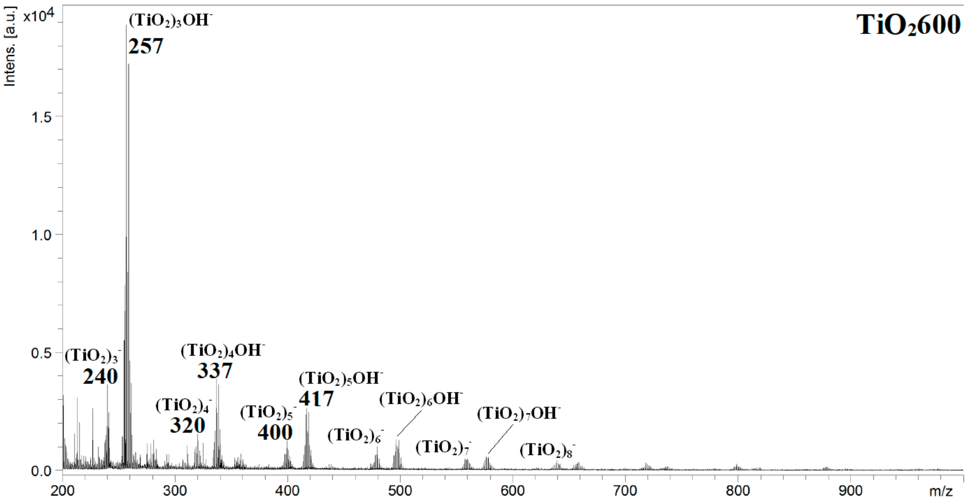

(f)

Figure S2. LDI mass spectra of TiO_2 samples, obtained in the negative ion mode, in the *m*/*z* range 200-1000, (a) $TiO_2$100, (b) $TiO_2$200, (c) $TiO_2$300, (d) $TiO_2$400, (e) $TiO_2$500, (f) $TiO_2$600.

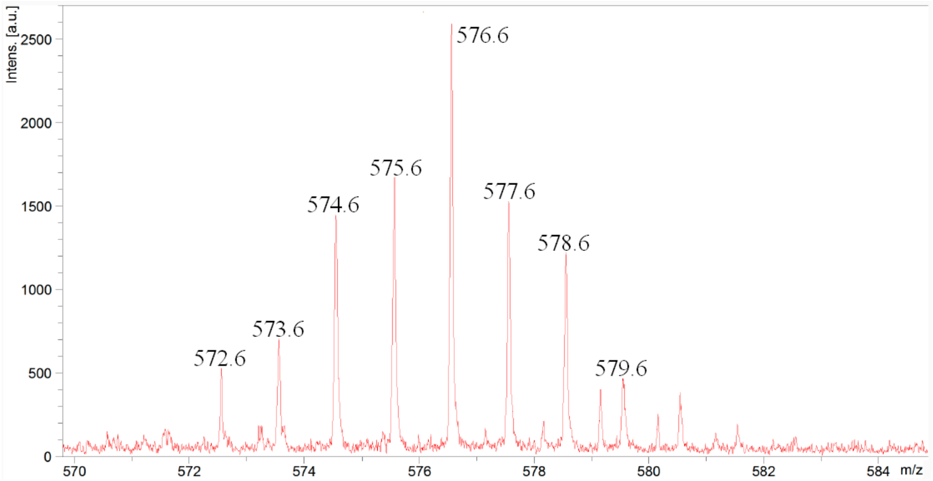

Figure S3. The obtained isotope pattern of $(TiO_2)_7OH^-$ ion, shown as a representative example. There is no doubt that we deal with the $(TiO_2)_7OH^-$ ion and not with the $(TiO_2)_7H_2O^-$ one (https://www.sisweb.com/mstools/isotope.htm).

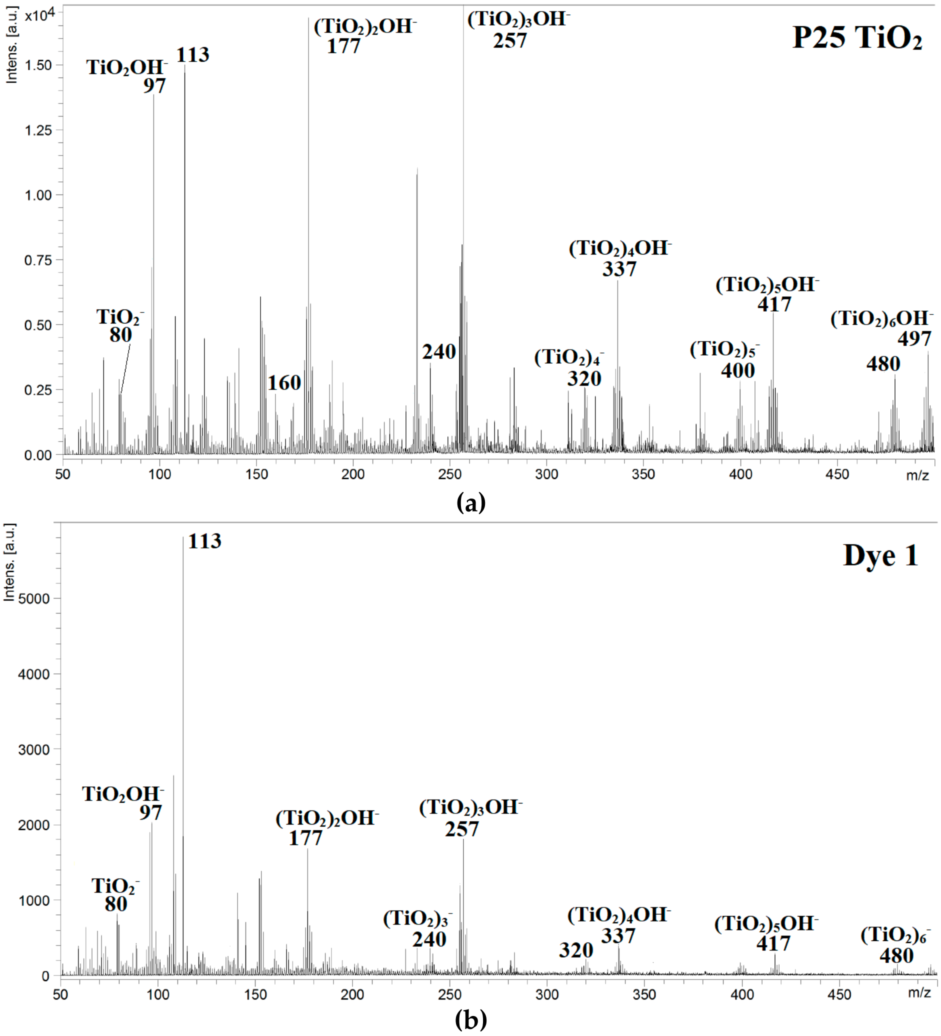

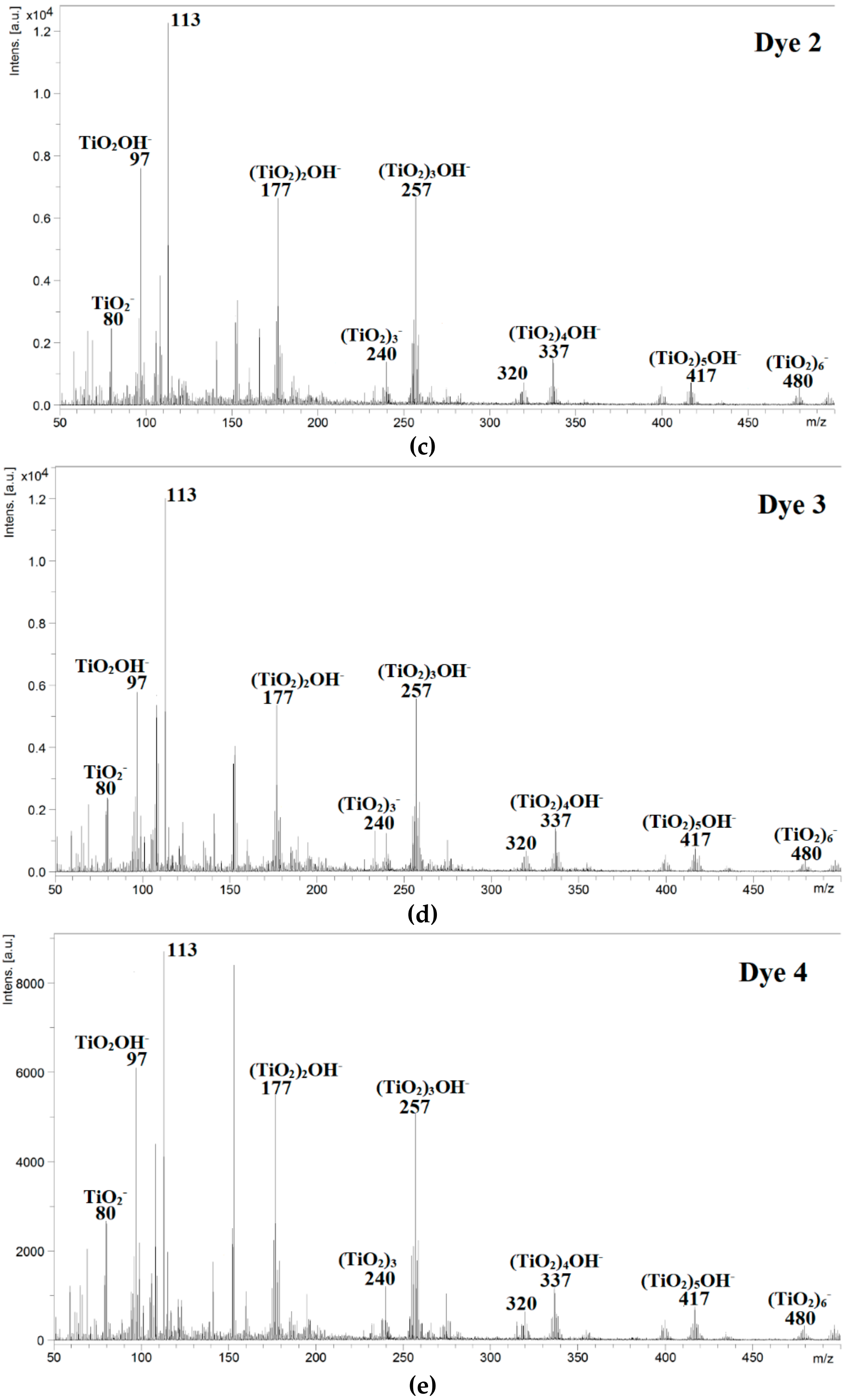

Figure S4. LDI mass spectra of the unmodified P25 TiO_2 and dye-modified samples, (a) P25 TiO_2, (b) Dye 1, (c) Dye 2, (d) Dye 3, (e) Dye 4.

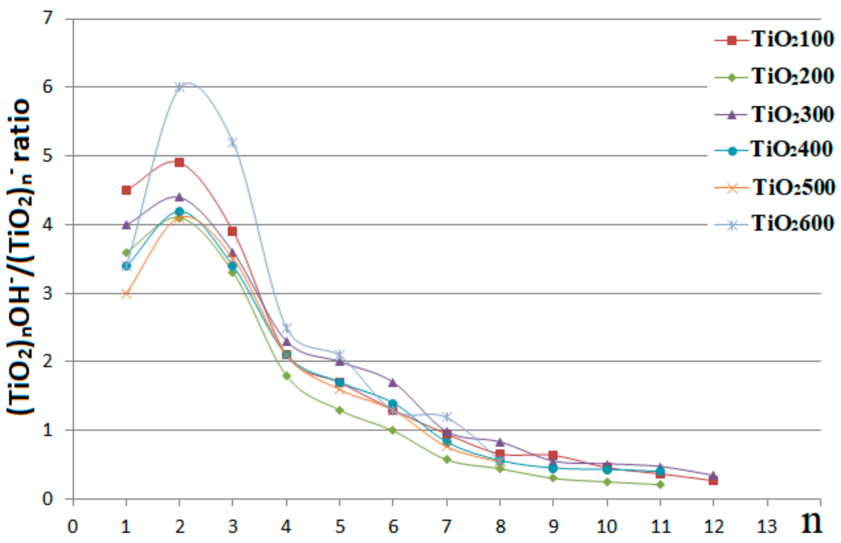

Figure S5. The breakdown plots of the $(TiO_2)_nOH^-/(TiO_2)_n^-$ ratio against the n number obtained for heated TiO_2 samples.

Since for TiO_2500 and TiO_2600, the ions at high *m/z* range are low abundant – Figure S2, the ratios for these ions are not included in the plots. The obtained plots are similar, however, not identical. Some of the plots can be used for the comparison/differentiation of the TiO_2 samples, e.g., the TiO_2100 and TiO_2600 plots are different than other plots for n≤4, the TiO_2200 plot is different than other plots for n>4, however, no comparison of the hydroxylation degree can be performed. For example the hydroxylation degree of TiO_2500 and TiO_2600 is similar, however, the latter plot, for n = 1-3, has a significantly different course than the former plot; for n ≥ 3, the $(TiO_2)_nOH^-/(TiO_2)_n^-$ ratios obtained for TiO_2200 have the lowest values. It is worth adding that TiO_2200 has very specific properties, e.g. different UV absorption than the other TIO_2 samples, due to the carbon deposit [25]. However the carbon deposit does not absorb the IR light, therefore it does not affect the FTIR spectrum shown in Figure 1 in the manuscript. In other words, not only hydroxylation degree significantly affects the $(TiO_2)_nOH^-/(TiO_2)_n^-$ ratio.

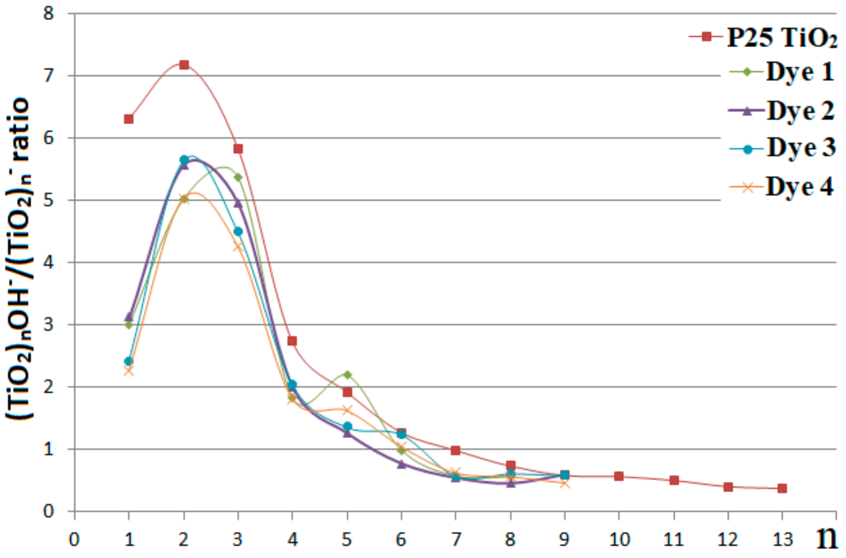

Figure S6. The breakdown plots of the $(TiO_2)_nOH^-/(TiO_2)_n^-$ ratio against the n number obtained for the unmodified P25 TiO_2 and dye-modified samples.

It is clear that for n = 1-3, the plot obtained for the unmodified P25 TiO_2 samples has a significantly different course than the other plots which have similar cousers. Therefore,

if TiO_2 samples differ only by the hydroxylation degree, the $(TiO_2)_nOH^-/(TiO_2)_n^-$ ratio depends on the free hydroxyl group population and can be used for comparison of the hydroxylation degree of TiO_2 samples.

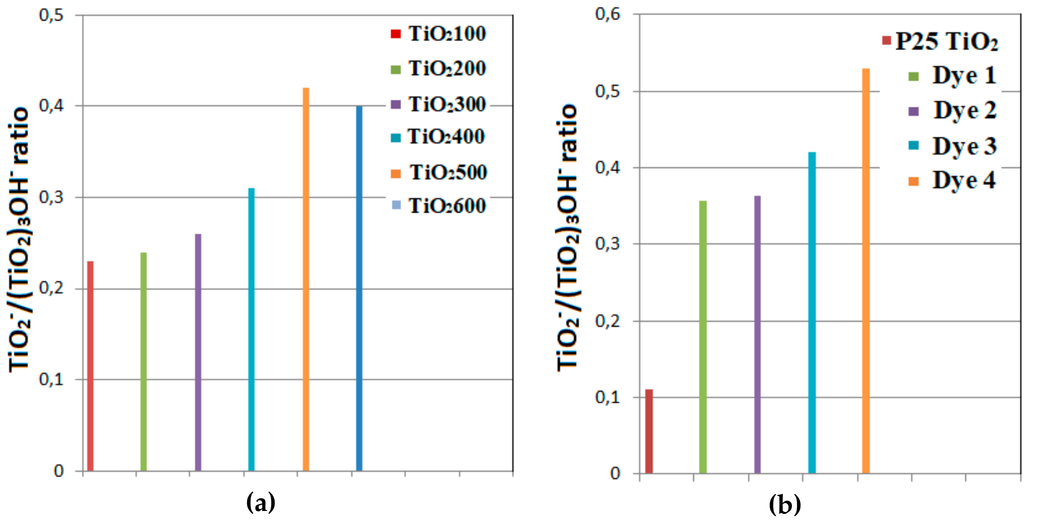

Figure S7. The $TiO_2^-/(TiO_2)_3OH^-$ ratios obtained for heated TiO_2 samples (a), unmodified (P25 TiO_2) and dye-modified samples (b).

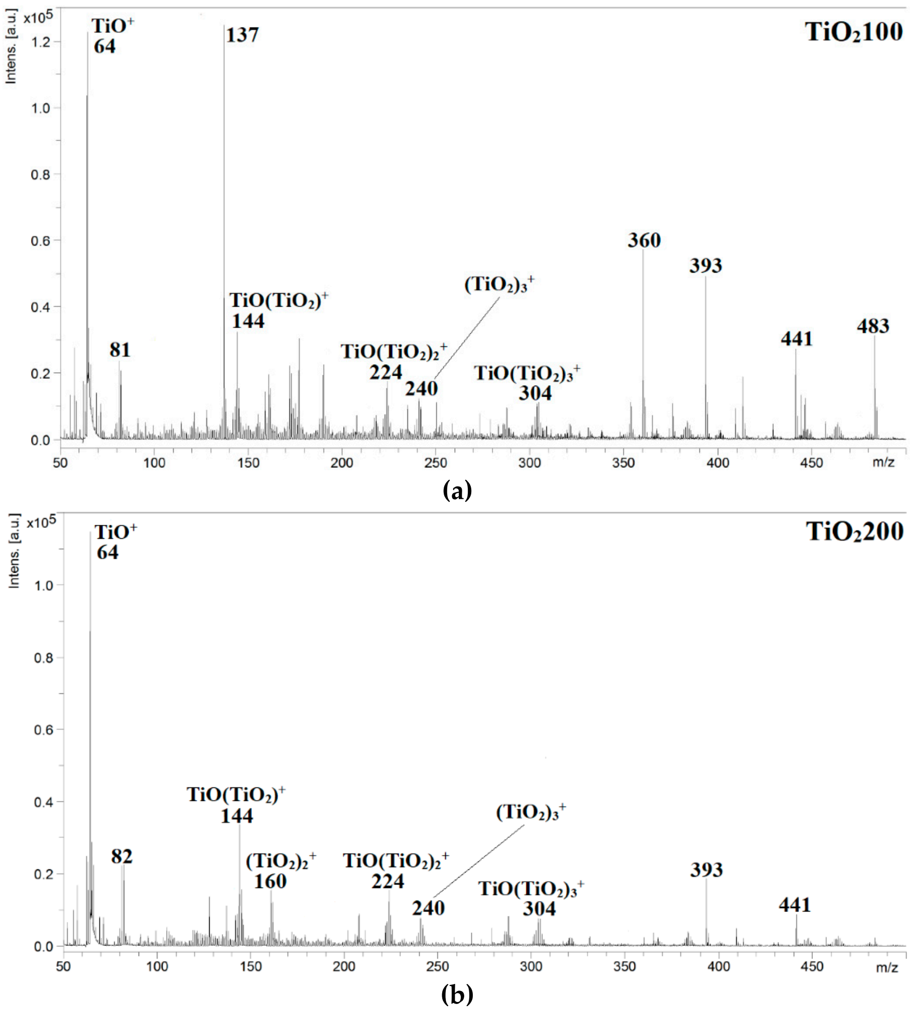

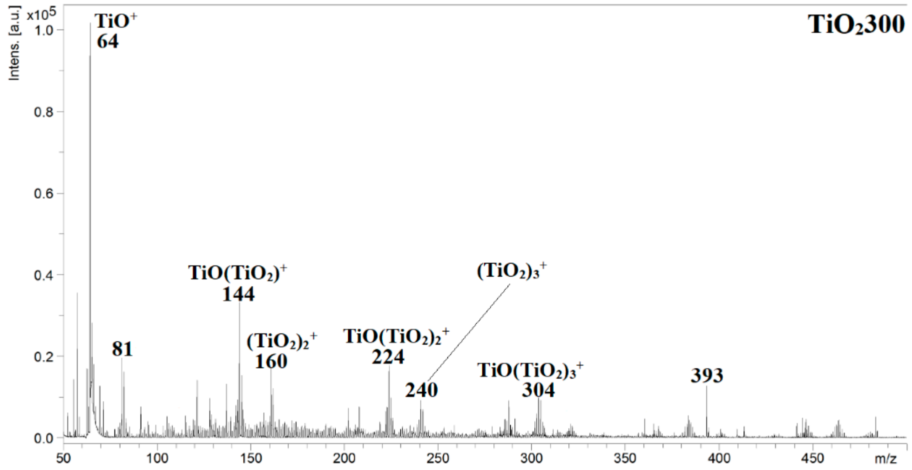
TiO2300
Intens. [a.u.]
x10^5
TiO+
64
81
TiO(TiO2)+
144
(TiO2)2+
160
TiO(TiO2)2+
224
(TiO2)3+
240
TiO(TiO2)3+
304
393
m/z

(c)

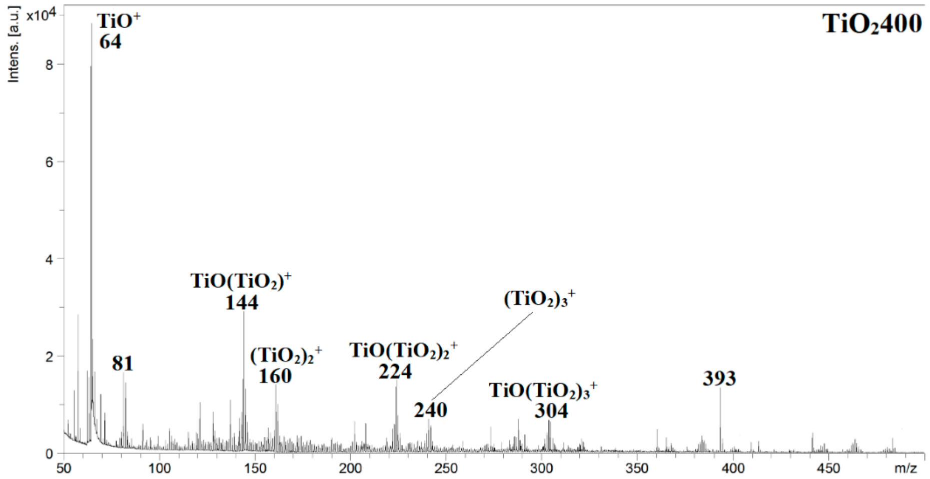
TiO2400
Intens. [a.u.]
x10^4
TiO+
64
81
TiO(TiO2)+
144
(TiO2)2+
160
TiO(TiO2)2+
224
(TiO2)3+
240
TiO(TiO2)3+
304
393
m/z

(d)

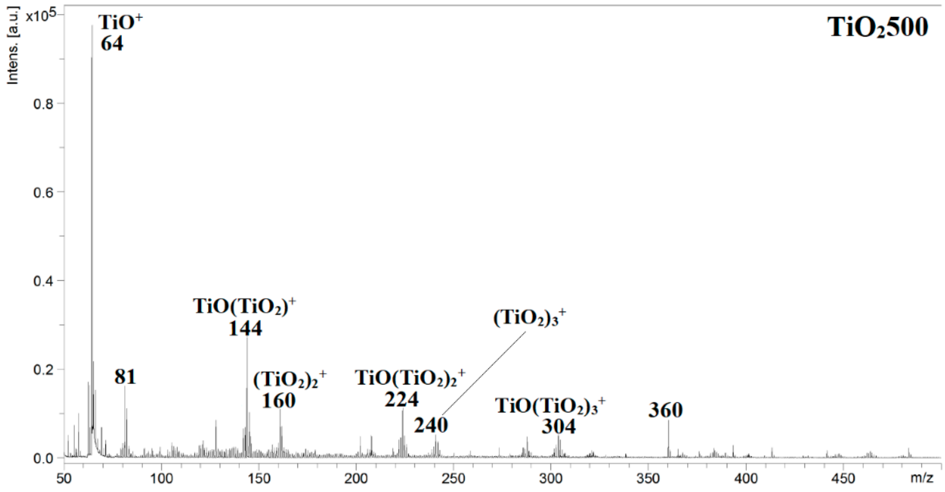
TiO2500
Intens. [a.u.]
x10^5
TiO+
64
81
TiO(TiO2)+
144
(TiO2)2+
160
TiO(TiO2)2+
224
(TiO2)3+
240
TiO(TiO2)3+
304
360
m/z

(e)

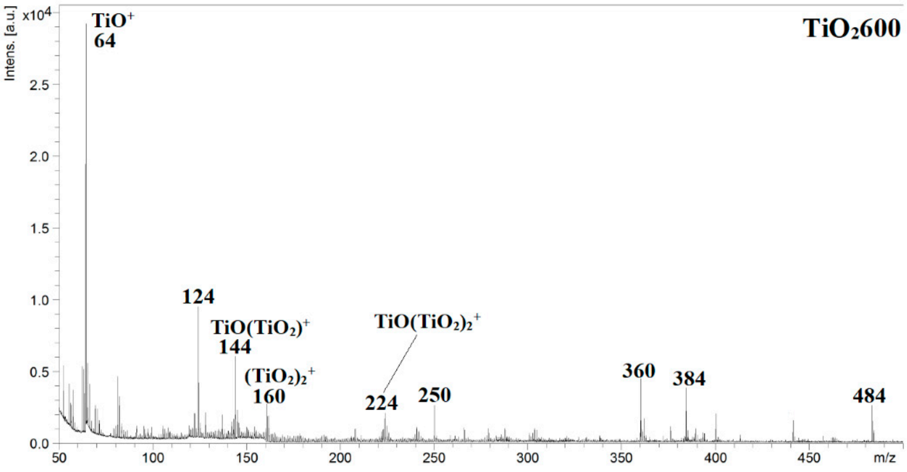

(f)

Figure S8. LDI mass spectra of TiO_2 samples obtained in the positive ion mode, (a) $TiO_2$100, (b) $TiO_2$200, (c) $TiO_2$300, (d) $TiO_2$400, (e) $TiO_2$500, (f) $TiO_2$600.

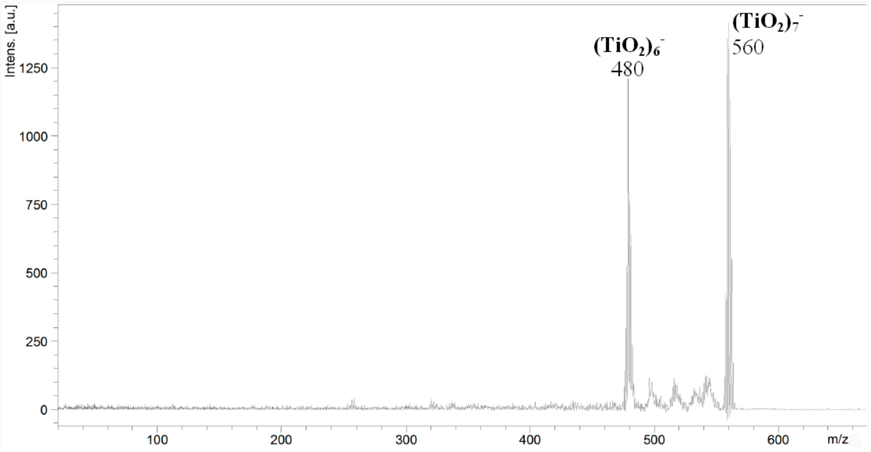

(a)

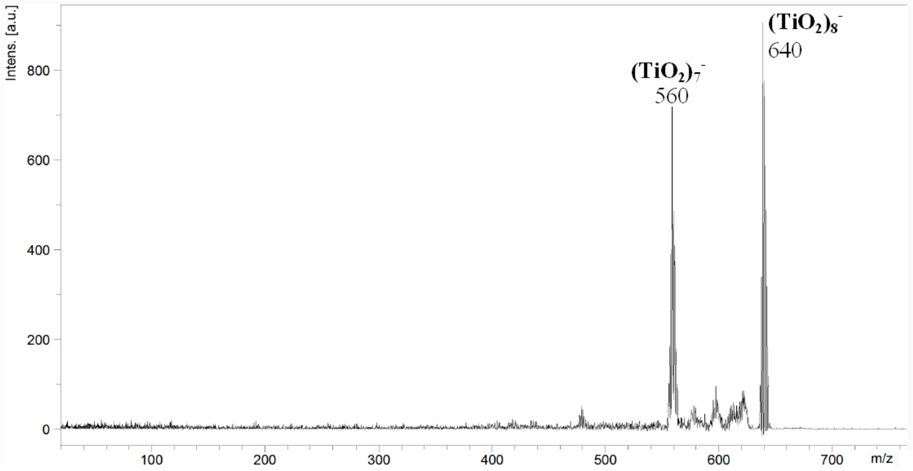

(b)

Figure S9. Exemplary LIFT mass spectra of $(TiO_2)_n^-$ ions. The selected parent ions were $(TiO_2)_7^-$ at *m/z* 560 (a) and $(TiO_2)_8^-$ *m/z* 640 (b). Because of a wide window, the selected ion beam and as a consequence the signals of all ions, contained isotope signals.

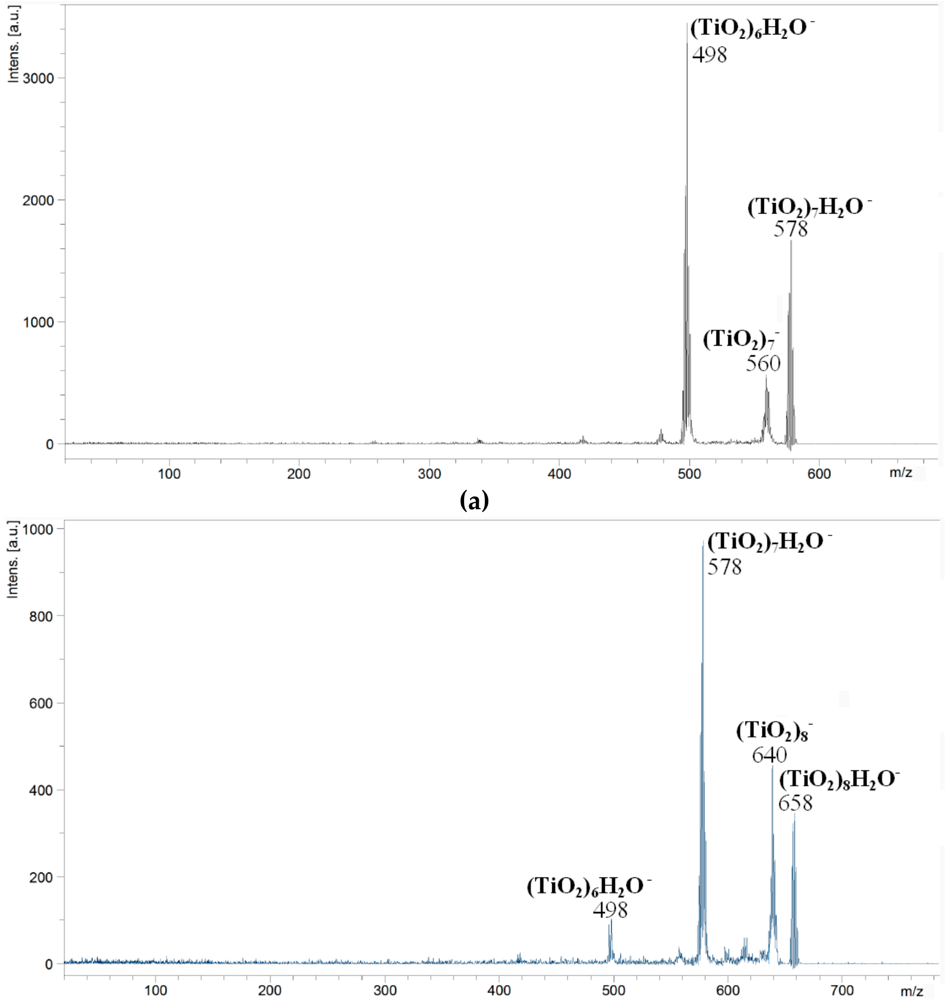

(a) **(b)**

Figure S10. Exemplary LIFT mass spectra of $(TiO_2)_nOH^-$ ions. The selected parent ions were $(TiO_2)_7OH^-$ at *m*/*z* 577 (a) and $(TiO_2)_9OH^-$ *m*/*z* 657 (b). Because of a wide window, the selected ion beam and as a consequence the signals of all ions, contained isotope signals.
